# Physiological assessment of the fetal body using MRI: current uses and potential directions

**DOI:** 10.1259/bjr.20221024

**Published:** 2023-07-05

**Authors:** Trevor Gaunt, Magdalena Sokolska

**Affiliations:** 1 Department of Radiology, University College London Hospitals NHS Foundation Trust, London, United Kingdom; 2 Department of Medical Physics and Biomedical Engineering, University College London Hospitals NHS Foundation Trust, London, United Kingdom

## Abstract

MRI assessment of the fetus using fast *T_2_*-weighted sequences is well established in defining alterations in fetal anatomy and structure, as a biomarker for disease, and in some cases prognostication. To date, the physiological assessment of the fetus using advanced sequences to characterise tissue perfusion and microarchitecture has largely been unused. Current methods of assessing fetal organ function are invasive and carry inherent risk. Therefore, the identification of imaging biomarkers of altered fetal physiology, and correlation with postnatal outcome, is attractive. This review describes the techniques which show promise for such a task and potential future directions.

## Introduction

Estimation of fetal organ function currently relies on the anatomical characteristics of ultrasound or MRI, or via invasive testing with amniocentesis or direct sampling. Anatomical assessment of the fetal body with MRI is well established. Fast *T*_2_-weighted spin echo and gradient echo sequences provide excellent spatial and contrast resolution between the fetus, the surrounding amniotic fluid and structures of pregnancy. Bowel configuration can be easily identified and assessed thanks to the T1 shortening effect of meconium, and T2* can be used to identify hemorrhage and calcified bone in the skeleton. However, structural scans do not provide quantifiable imaging characteristics pertaining to fetal tissue properties. Such characteristics could be correlated with postnatal biomarkers of disease and allow the prediction of outcomes in early childhood. To date, advanced MRI sequences used for the physiological study of the developing fetus have mostly focused on applications in the cardiovascular and central nervous systems. However, there is an emerging body of research to support their use in the development of other fetal organs. This review describes chosen advanced MRI sequences, outlines their current use in the physiological assessment of the fetus, and provides potential future developments.

## Techniques

### T2*-weighted imaging and T2* mapping

The contrast in T2*-weighted imaging arises from the dephasing of transverse magnetization due to local susceptibility gradients, resulting in local field inhomogeneities. It is achieved by the acquisition of gradient-echo images with long TE and TR. For example, deoxygenated hemoglobin is paramagnetic and causes local T2* relaxation time shortening. This results in contrast between areas containing paramagnetic deoxyhemoglobin and those containing diamagnetic oxyhemoglobin, the latter returning higher signal and appearing brighter on a T2*-weighted scan. This phenomenon is termed the Blood Oxygen Level Dependent (BOLD) effect and can be used to assess tissue response to maternal hyperoxia.^1^ Similarly, paramagnetic iron within tissue causes local field inhomogeneity and shortening of the T2* relaxation time leading to accelerated signal decay.

**Figure 1.**
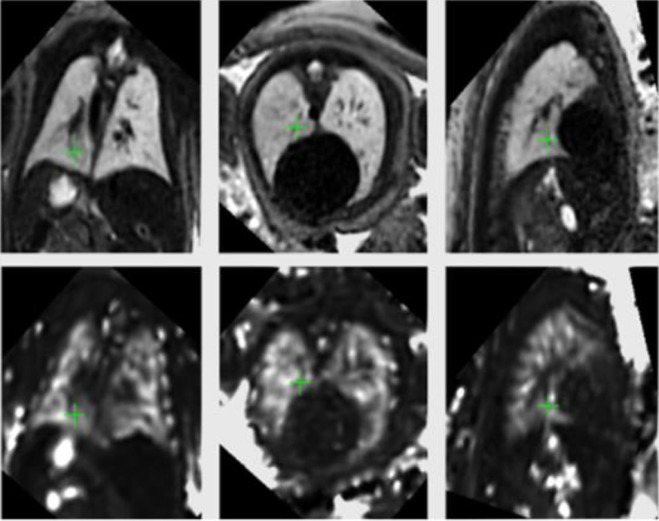
3T Coronal T2, axial and sagittal T2 (top row) and T2* (bottom row) aligned maps of a healthy fetus at 35 weeks gestation. Reproduced from Avena-Zampieri et al 2022.^4^

T2* mapping refers to a voxel-wise calculation of T2* relaxation time from multiecho data, via acquisitions of T2*-weighted images at a range of echo times. The longer the echo time, the greater the degree of spin dephasing, resulting in a lower signal; this can be modelled as a mono-exponential decay. The choice of TEs for T2* mapping should be dictated by the range of clinically expected values. The literature recommends starting from the shortest echo time possible, and echo spacing of 1 ms.^2^ T2* maps can be particularly useful in the assessment of iron overload^3^ and multiecho T2*-weighted images can also be reviewed qualitatively^4^ (Figure 1).

### Chemical shift encoded MRI

Chemical shift encoded MRI (CSE-MRI) exploits chemical shift, the small difference in resonant frequencies between protons in different molecular environments. The simplest implementation of CSE-MRI (Dixon) acquires two images at echo times where fat and water spins are in and out of phase. The signal contributions from fat and water add and subtract respectively, and allow computation of fat and water-only images and fat signal fraction [FSF = fat/(water+fat)]. To counteract some of the limitations of the initial Dixon method, the current CSE-MRI relies on multiple echo data to model the spectral complexity of fat, using algorithms such as IDEAL^5^ (iterative decomposition of water and fat with echo asymmetry and least squares estimation). IDEAL can be adapted to simultaneously model a T2* decay which allows for the calculation of a proton density fat fraction (PDFF)^6^ independent of the scanner and acquisition parameters, and can be utilised to estimate the amount of lipid content in a developing fetus.^7^

### Diffusion-weighted imaging and ADC mapping

Diffusion-weighted imaging (DWI) is sensitive to the random movement of a water molecules in tissue. DWIs can be acquired with a different amount of diffusion weighting through a change of b-value; a factor incorporating the amplitude and timing of diffusion-sensitising gradients. The apparent diffusion coefficient (ADC) can be quantified by a mono-exponential model fit to the data acquired at multiple b-values (including *b* = 0 s/mm^2^) resulting in a parametric map. The ADC is higher in free water, medium when water movement is hindered (i.e. outside of cells) and lowest when this movement is restricted (i.e. within cells). DWI and ADC can therefore potentially be used to estimate micro-level changes in tissue function due to pathology.

DWI in the fetal body is usually acquired using b-values between 0 and 700 s/mm^2^ to provide sufficient diffusion sensitization whilst preserving signal-to-noise ratio (SNR).^8^ Images acquired with higher b-values are characterised by the lower SNR and require longer echo times, which can additionally contribute to signal loss and distortions, particularly from fetal motion.

### Intravoxel incoherent motion

Intravoxel incoherent motion (IVIM) denotes a quasi-random distribution of translational movement within a voxel.^9^ The data acquisition is similar to DWI; however, it requires sampling a broad range of b-values from low (below 100 s/mm^2^, including 0) to high (above 300 s/mm^2^).

The measured signal attenuation is assumed to be driven by the sum of microperfusion (collective capillary blood flow that is considered random) and tissue diffusion. This is modelled as biexponential decay, a weighted sum of two exponents. The microperfusion component contribution to the IVIM signal is greatest within the low b-value ranges and can be quantified as the pesudodiffusion coefficient D*. The diffusion component contribution to the IVIM signal is greatest within the high b-value ranges and can be quantified as the diffusion coefficient D. The percentage of a voxel volume occupied by capillaries (the weighting factor) is denoted by f. D* and f could therefore act as potential biomarkers for tissue vascularity.

The range of b values chosen for the IVIM acquisition should be optimised to best estimate D, D* and f for the tissue and pathology in question. This can be achieved via computer experimental design (CED) simulations, with the addition of Rician noise and minimalization of parameter estimation variance.^10^ Newer approaches incorporate the ability to classify different subject groups, such as normal and abnormal tissues.^11^

## Challenges

T2* mapping, CSE-MRI, ADC, and IVIM require multiple acquisitions of the same anatomy with varying echo time or diffusion gradient weighting. This is especially challenging in the presence of fetal motion and maternal respiration during acquisition, resulting in the misalignment of acquired data. These effects can be minimised to an extent during acquisition, and with motion correction post-processing.

Short acquisitions such as multislice echo planar imaging (EPI) can be used for T2* mapping and markedly reduce the effect of motion during slice acquisition,^12^ enabling larger coverage. Alternative solutions include single-slice^13^ and 3D breath-hold acquisitions^14^ or acquiring data sets as a 3D stack-of-radial multigradient echo Dixon sequences, which are less sensitive to motion and can be acquired during free breathing.^15^ Although promising, this last development is not yet available commercially and requires more validation before it becomes commonplace.

Image frames with intra-acquisition motion resulting in signal loss or loss of fetal body definition must be identified and removed before further processing,^16^ and inter-acquisition motion corrected using a rigid transformation. If the data are acquired in multiple orientations, a processing method called slice-to-volume registration can be performed. This approach was shown useful in fetal brain and quantitative placental T2* mapping, but can be challenging for correction of fetal body motion due to simultaneous interslice and non-rigid motion.^17^

## Applications

## Lungs

### T2* imaging

Evaluation of the fetal lungs is critical for risk stratification in the context of pathology which might lead to neonatal pulmonary hypoplasia, and the morbidity and mortality associated with prolonged pulmonary hypertension. Predicting neonatal outcomes in conditions such as intrauterine growth restriction or twin-twin transfusion syndrome; or any process resulting in oligohydramnios such as premature rupture of membranes and fetal urinary tract obstruction; would be an invaluable tool in prenatal counseling.

Observed/expected lung volume ratio on MRI^18^ has been useful in correlating outcome following prenatal intervention in congenital diaphragmatic hernia.^19^ Moreover, T2 signal has been shown to change with gestational age and is associated with poorer outcomes in CDH when normalised against the liver signal^20,21^ suggesting that the microarchitecture of the lung might be a suitable target for a functional imaging biomarker to predict post-natal function.

Khen-Dunlop et al examined 38 healthy fetuses to see whether the BOLD response could be detected in fetal lungs. Maternal hyperoxia was induced for 5 min before the BOLD sequence, with R2* (1/T2*) evaluated by average signal intensity for both normoxic and hyperoxic periods. They demonstrated a significant BOLD response after maternal hyperoxia in the fetal lungs with a mean R2* decrease of 12.1±2.5% (*p* < 0.001) confirming oxygen uptake.^22^ The BOLD response could therefore be used in addition to the lung volume for a better prediction of postnatal respiratory status, although there are no clinical studies reported in the current literature.

Using a modified IDEAL algorithm, Sethi et al^14^ showed an increasing trend in T2* values with gestation until 30 weeks, in nine uncomplicated pregnancies between 28 and 38 weeks. This has since been replicated by Avena-Zampieri in 87 motion corrected data sets from healthy fetuses, using a multiecho single-shot EPI sequence at 3T.^23^ The increase in T2* may be due to an increase in metabolic activity, and therefore a change in the proportion of oxygenated and deoxygenated hemoglobin in the lung capillary bed during maturation.

### DWI/ADC and IVIM

**Figure 2.**
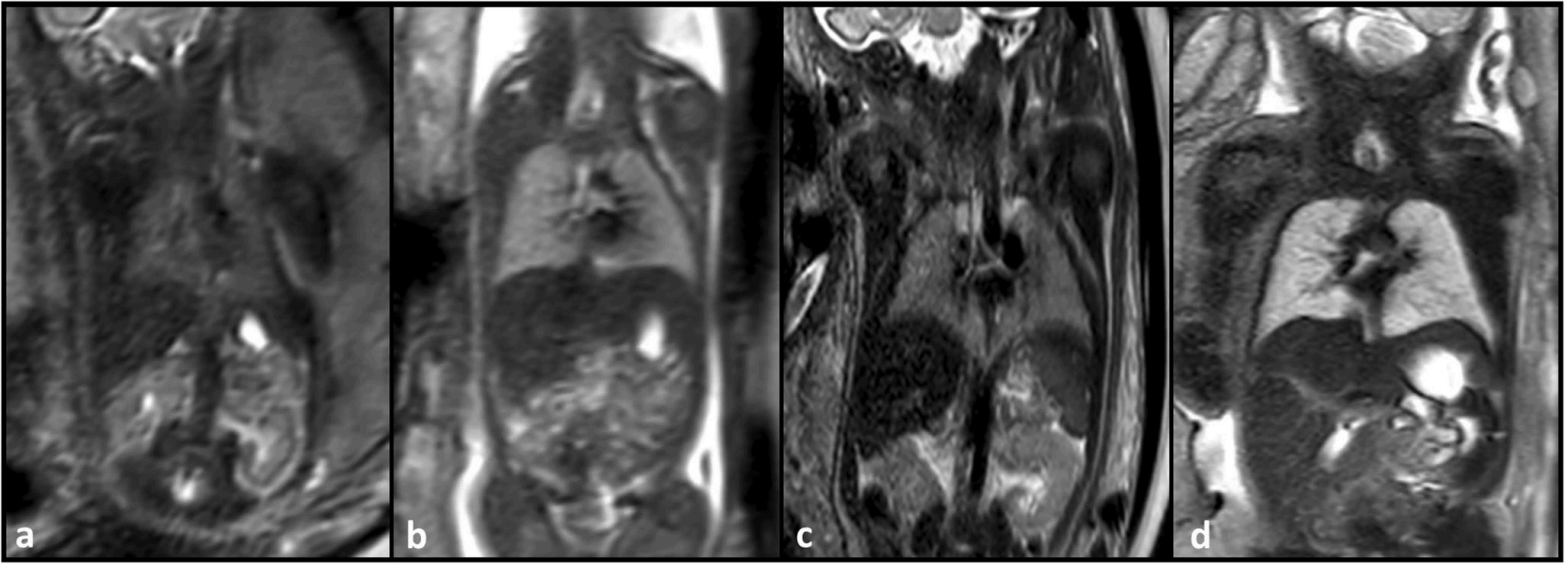
Coronal *T*_2_-weighted fetal MR images (a–d) of two fetuses with premature rupture of membranes and subsequent anhydramnios and pulmonary hypoplasia (**a, c**) at gestation weeks 23 (**a**) and 32 (**c**), and of two fetuses with normal lung development (**b, d**) at gestation weeks 23 (**b**) and 32 (**d**). The lungs in fetuses with premature rupture of membranes, anhydramnios, and pulmonary hypoplasia show hypointense T2 signal (**a, c**) compared to the controls at the same gestational age (**b, d**). Furthermore, T2 signal increases throughout gestational age (**b, d**). Reproduced from Prayer et al.^8^

Several groups have demonstrated the feasibility of using diffusion-weighted imaging to assess the fetal lung, some with conflicting results over how ADC is related to lung maturity.^18,24,25^ However, Afacan et al^26^ showed lung ADC values are significantly associated with gestational age, increasing between 16 and 27 weeks, before plateauing around 27 weeks using 6 b-values (*p* < 0.01). Interestingly, they showed ADC value did not show a significant relation with gestational age, compared to when only *two* b-values were used. Moradi et al compared lung/liver ADC ratio (LLADCR) and lung/muscle ADC ratio (LMADCR) in 49 IUGR and 58 non-IUGR fetuses at 3T, finding both were significantly lower in the latter group. It is not completely clear how the microstructure of the lung is related to ADC values, but it is likely that the increasing amount of fluid in the airways and alveoli, and the increasing density in pulmonary vasculature within the developing lung play a role^27,28^ (Figure 2). Nevertheless, children born prematurely and with IUGR possess a deficiency in lung function relative to their normal weight-for-gestation peers by school age. This suggests the signal changes observed through fetal life support a progression in maturation which if disturbed, have sequelae for early years respiratory function.^29,30^

Jakab et al^30^ tested the within-subject repeatability of f, D and D* in the placenta, and the fetal lung and liver at 1.5T and 3T, using 16 different b- values between 0 and 900 s/mm^2^. They found f, D, and D* could be measured in 15 pregnancies with within-subject test-retest variability in the range of 14.4–20.4% for f, 12.2–14.1% for D, and 16.8–25.3% for D*. However, the perfusion and diffusion parameters depended heavily on image quality, fetal and maternal movements, and fetal-specific image post-processing. Not unsurprisingly, the hila regions demonstrated a greater f than the lung periphery. They also found a global reduction in microvascular perfusion in the hypoplastic lung in a fetus with congenital diaphragmatic hernia, and within a CPAM in a second fetus.^31^

The same group showed an increase in perfusion fraction with maturity in the lungs of 33 pregnancies between 17 and 36 weeks gestational age.^16^ Furthermore, Ercolani et al^32^ investigated the use of IVIM to study the microstructure changes in the fetal lung. Thirty-four normal pregnancies were divided into two groups according to gestational age of between 21–29 weeks and 30–39 weeks. Multiple diffusion-weighted echo planar imaging sequences were performed with a range of b- values 0, 10, 30, 50, 75, 100, 200, 400, 700, and 1000 s/mm^2^. Mean values of perfusion fraction were significantly higher in fetuses between 30 and 39 weeks gestational age, suggesting f shows potential as a marker of pulmonary maturation.^32^

## Liver and gastrointestinal tract

### T2* imaging

Clinical outcomes in conditions such as neonatal and congenital haemochromatosis, and neonatal haemosiderosis are poor without appropriate treatment, leading to hepatic failure and infantile death. T2* values for fetal liver evaluation might therefore assist in the early diagnosis of iron overload syndromes^13^ prompting early intervention or support for late termination.

Goitein et al^13^ defined normal third trimester T2* relaxation values in 46 fetuses using a 16 echo T2* sequence at 1.5T. The average reported T2* was 19.7 ± 7.4 ms with excellent inter- and intra-observer agreement. The study in question was prompted following a clinical MRI for a fetus with suspected neonatal hemachromatosis; this fetus had a liver T2* between 8 and 9 ms^13^.

Sethi et al^14^ studied the T2* relaxation times of various organs within the healthy developing fetus. They found no significant change in T2* relaxation time in the liver, but there was a significant reduction in T2* relaxation time in the spleen. This is likely associated with progressive fetal extra medullary hematopoiesis, and the increasing amount of paramagnetic iron contained within the spleen throughout gestation.^33–36^ Although the liver is hematopoietic, the spleen is more responsible for hematopoiesis during the third trimester.^33^

Abaci Turk et al studied T2* relaxation time in 38 pregnancies during Braxton Hicks contractions using a multiecho EPI sequence at 3 T^37^. Regions of interest for the uterus and fetal organs were drawn in the reference frame using ITK-SNAP.^38^ In this small population, they demonstrated a reduction in placenta, brain and liver T2* during Braxton Hicks contractions suggesting transient decreases in both placental and fetal organ oxygenation.^37^ Other work has shown a T2* reduction in placental failure, intrauterine growth restriction and small for gestational age fetuses,^39^ as well as changes in the BOLD effect during maternal hyperoxygenation.^40^ This suggests that T2* might be useful for assessing the degree of fetal organ hypo-oxygenation in the future.

### DWI/ADC and IVIM

Jakab et al^31^ demonstrated a sharp decrease in microvascular perfusion fraction in the fetal liver with gestational age, with an f value 30% lower in the third trimester compared to the second.^31^ The fraction of arterial blood shunted *away* from the liver increases with gestational age, suggesting changes in microvascular perfusion fraction with gestational age may indirectly reflect the redistribution of fetal circulation. Perfusion fraction could therefore represent a surrogate biomarker for microvascular maturation, with the potential to be used in combination with changes in T2* to reflect the degree of hypo-oxygenation in conditions such as IUGR and placental failure.

## Kidneys and genitourinary tract

### T2* imaging

The kidneys have been the most common target for the development of an MRI biomarker of function. Similar to the lungs, estimaton of fetal renal function currently relies on the greyscale assessment of fetal kidneys and amniotic fluid volume on ultrasound. Fetal serum β2-microglobulin (fsβ2M) following cordocentesis has been shown to correlate with post-natal renal function in fetuses with posterior urethral valves treated with vesicoamniotic shunts. As this carries the risks of any prenatal intervention, a less invasive biomarker to predict postnatal renal function is desirable.

One preliminary study suggested the BOLD effect could be used to estimate the degree of renal ischaemia in the context of prenatal urinary tract obstruction, and furthermore as a biomarker for postnatal renal function and counselling.^41^ Chalouhi et al described the BOLD effect in a small population of rats with iatrogenic obstructive uropathy. In the obstructed kidney, the BOLD effect fell suggesting a transient *rise* in oxygen consumption and renal perfusion, followed by a gradual rise in the BOLD effect suggesting a *fall* in oxygen consumption and perfusion. After 6 days, the BOLD effect fell again suggesting ischaemia and non-perfusion. Interestingly, the contralateral kidney experienced the opposite effects with a reduced BOLD effect throughout the experiment in keeping with compensation.

Moreover, Sethi et al found no change in the T2* relaxation time of the kidneys with gestational age, unlike in the spleen and lungs.^14^ Sørensen et al studied the BOLD effect in a small number of healthy pregnancies between 28 and 34 weeks gestation, finding that there was around a 6% increase in BOLD MRI signal in the fetal kidney during maternal hyperoxia.^1^

### DWI/ADC and IVIM

**Figure 3.**
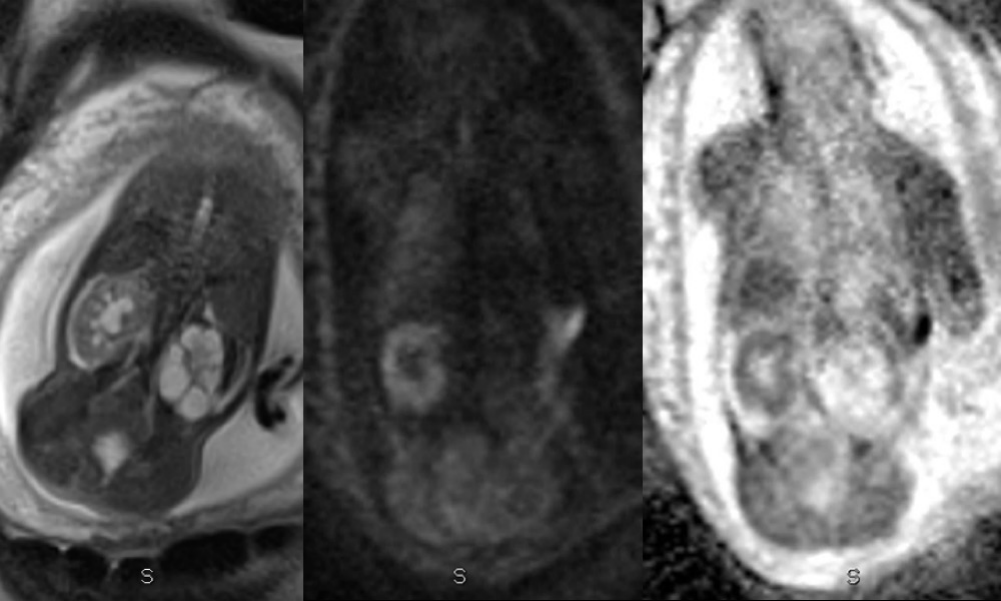
Coronal T2 HASTE sequence shows a right kidney with pelvicaliceal dilatation in a fetus at 28 weeks. The renal parenchyma on the corresponding DWI sequence returns high signal, with low values on the ADC map suggesting restriction of free water diffusion. In contrast, there is no corresponding high DWI signal returned from the left-sided multicystic dysplasic kidney, suggesting the absence of normal renal tissue.

Normal fetal kidneys demonstrate restriction of free water diffusion similar to that of an adult kidney. This is particularly useful in identifying the location of ectopic kidneys, and for differentiating between severe hydronephrosis and multicystic dysplastic kidney (Figure 3), which may not be clear at a prenatal ultrasound. Additionally, the corresponding ADC value shows potential as a biomarker for renal function. A number of groups have suggested DWI/ADC assessment of the fetal kidneys is feasible, but with conflicting conclusions.

Witzani et al found ADC values decreased throughout gestation but plateaued at 28 weeks^8^. Manganaro et al studied 88 fetuses between 17 and 40 of gestational age divided into six groups. Likewise, they found an inverse relationship between ADC values and increasing gestational age.^42^ This can be attributed to the ongoing development of the functional unit of the kidney, with an increase in the size and number of nephrons, and therefore tubular flow and parenchymal blood flow through the gestational period and beyond into childhood. Furthermore, the same group showed ADC values correlate with the amount of functioning renal tissue.^43^ Renal parenchyma showed an increase in ADC value compared to normal kidneys in the context of urinary tract obstruction and dysplastic renal diseases. Similarly, Ercolani et al showed IVIM perfusion fraction increased with gestational age.^32^

Conversely, Chaumoitre et al found no significant difference in renal ADC value in 51 fetuses between 23–38 weeks of gestational age. Interestingly however, they did find that the ADC of the donor twin was higher than that of the recipient twin in cases of twin-twin transfusion syndrome, with the difference appearing to be related to the severity of the syndrome.^44^

More recently, Faure et al evaluated whether ADC could predict postnatal renal function in 11 male infants with posterior urethral valves (PUV) using nadir creatinine and eGFR at 1 year.^45^ ADC values were measured within the renal parenchyma of both kidneys in both the coronal and axial planes, using the best image containing the whole kidney. A mean ADC value was calculated and compared to normal range of 1.1 to 1.8 mm^2^/s described by Chaumoitre et al.^44^ Two fetuses with enlarged hyperechoic kidneys, oligohydramnios and raised renal ADC values at 2.6 mm^2^/s and 2.86 mm^2^/s underwent termination of pregnancy, and had raised ADC values at 2.6 mm^2^/s and 2.86 mm^2^/s. Surviving fetuses had abnormal ADC values between 1.8 and 2.3 mm^2^/s. Each of these four had pulmonary hypoplasia and developed acute renal failure shortly after birth and had raised nadir creatinine (median 55 µmol l^−1^, range 30–65) and reduced nadir eGFR (median 42 mL/min). One of these four infants died at 6 months of age from renal insufficiency despite peritoneal dialysis. The five fetuses with normal ADC values had normal nadir creatinine (25 µmol l^−1^; range 23–48) and eGFR between 109-120 mL/min.

Although promising, the majority of these studies are in small populations of fetuses, using different scanner vendors and sequence parameters making them difficult to compare. Much larger studies to validate reference ranges for normal kidneys with post-natal renal function would be necessary before translating into clinical practice.

## Adipose and body composition

Fetal body composition can be altered in growth-restricted fetuses, and those of mothers with diabetes and macrosomia.^15,46^ Adipose tissue is easily demonstrated with MRI making quantification of fat volume possible and in turn enabling its monitoring of fetal health through its development.

Anblagan et al compared the subcutaneous fat volume in 14 fetuses of mothers with pre-gestational diabetes against 12 non-diabetic controls at two gestation ages: 24 and 34 weeks.^47^ Fetal fat volume was determined from a *T*_1_-weighted water-suppressed image mask and calculated as a proportion of total fetal volume estimated from manual segmentation of the non-suppressed image. Fetal weight was then estimated from the volumes using densities of 0.9 kg l^−1^ for fat and 1.064 kg l^−1^ for non-fat tissue at term. Fat content was similar in both groups at 24 weeks but increased significantly in those of diabetic mothers at 34 weeks with a mean of 1090 ± 414 cm^3^ compared to 541 ± 348 cm^3^. Moreover, Berger-Kulemann et al found fetuses of mothers with well-controlled diabetes did not have thicker subcutaneous fat layer compared to controls.^48^ Fat volume estimation may therefore act as a surrogate marker for altered fetal metabolism which could result in the development of a metabolic syndrome later in life.

Giza et al^46^ used a modified 3D two-point Dixon technique to assess lipid volume, lipid/fetal volume ratio and signal fat fraction of 17 fetuses of mothers with the range of the body-mass-index (BMI range 19.2–52.2 kg/m^2^). They found a strong positive correlation between gestational age in all three assessed MRI parameters (lipid volume from 4 to 48 ml, lipid/fetal volume ratio 0.3–1.8% and FSF from 10 to 24% between 30 and 34 weeks of gestation age) in trunk subcutaneous tissue.^46^ Strobel et al^15^ compared three cohorts: healthy, those with gestational diabetes, and fetal growth restriction, using a prototype free-breathing 3D stack-of-radial multiecho gradient echo CSE-MRI. This allowed the quantification of the hepatic proton-density fat fraction (PDFF), in addition to the maternal visceral and both maternal and fetal adipose tissue volumes in a single data set. In this study, fetal adipose tissue volume positively correlated with gestational age and maternal pancreatic, but not hepatic, PDFF and with infant birth weight z-score. Additionally, the authors found that fetuses of mothers with gestational diabetes had higher volumes of adipose tissue and higher hepatic PDFF compared to fetuses from other groups, meaning PDFF could potentially be developed as a prenatal imaging biomarker for childhood fatty liver disease.

## Potential directions

The *in vivo* use of hyperpolarised MRI contrast agents for metabolic assessment of the fetoplacental relationship is a new field, with only animal models cited in the literature. However, the technique may be deployed for the non-invasive assessment of fetoplacental metabolism and nutrient transport. The hyperpolarization process increases the signal of a metabolic substrate by 10,000–1,000,000 fold, meaning a hyperpolarised substrate “label”, and its metabolites, can be imaged. Friesen-Waldner et al demonstrated the feasibility of using hyperpolarised [1–13C] pyruvate in a guinea pig model. *T*_1_-weighted images were obtained after maternal injection of a hyper polarised [1–13C] pyruvate solution, with pyruvate and lactate signals demonstrated in fetal livers at 10 s and 20 s, respectively. Hyperpolarised 13C MRI, therefore, might be used in the assessment of abnormal fetoplacental transport, implicated in IUGR, macrosomia, and gestational diabetes.^49^ More work of course would be necessary to assess the safety profile of any hyperpolarised substrates prior to administration in pregnancy and the development of multinuclear body coils.

Magnetic resonance spectroscopy (MRS) is well established for the assessment of the fetal brain, but recent developments in fetal metabolomics might allow for the study of amniotic fluid *in utero*. Currently, examination of fetal metabolites in the amniotic fluid requires invasive sampling via amniocentesis, which allows for the characterization of biomarkers of disease by NMR spectroscopy.^50–52^ However, this carries a risk to the pregnancy, and a non-invasive method would be preferable. MRS pulse sequences are relatively slow, and hence useful spectral data are hindered by fetal and maternal motion. Furthermore, spectral shift due to magnetic susceptibility interfaces and magnetic field drift over the large imaging volumes have impeded the development of fetal MRS imaging. Naila et al developed an amniotic fluid phantom, validated with NMR spectroscopy, which simulates the environment of a gravid uterus.^53^ With a more controlled environment, improvements in signal acquisition, voxel size reduction and the use of various higher-order shimming techniques would become feasible for MRS sequence optimisation, and is a promising step toward non-invasive *in utero* metabolomics.

Studies of reproducibility using techniques described in this review are scant and most research into reliability has concentrated on anatomical measurements, such as fetal lung volume in congenital diaphragmatic hernia. Some small studies show good reproducibility for other techniques, such as that described for f, D and D* in the fetal lungs.^30^ However, others show poor reproducibility, or no large studies of reproducibility have been conducted at all. Therefore, the ongoing development of motion correction post-processing algorithms and use of artificial intelligence in fetal MRI might improve reproducibility, or even negate the need to draw regions of interest at all for functional assessment in future.

## Conclusions

Functional assessment of the fetal body using prenatal MRI imaging biomarkers of disease shows huge potential for predicting postnatal outcomes. Advanced MRI techniques allowing the assessment of fetal body function are described in this review, and these are summarised below and in Table 1. Practice guidelines detailing MRI protocols and parameters have recently been published elsewhere in the literature.^54^

**Table 1.** Summary table of potential functional MRI biomarkers for fetal physiology.

Sequence	Organ	Use
T2* & BOLD	Lung Liver Kidney	T2* value increases with gestational age T2* value possible predictor of pulmonary hypoplasia relative to ipsilateral lung in CDH BOLD effect possible marker for oxygen uptake in the fetal lungs T2* value consistent through gestational age T2* value therefore biomarker for suspected neonatal hemochromatosis T2* value reduces during Braxton Hicks contractions and therefore potential biomarker for fetal organ hypoxia T2* value is reduced in iron overload syndrome T2* value consistent through gestational age BOLD effect in fetal renal ischemia^a^ and maternal hyperoxia
DWI & IVIM	Lung Liver Kidney	ADC value increases with gestational age according to most studies and when corrected against the liver ADC value potential imaging biomarker for lung hypoplasia and poor neonatal outcome Perfusion fraction increases with gestational age Global reduction in microvascular perfusion fraction in hypoplastic lungs in CDH fetuses Perfusion fraction decreases with gestational age and therefore potential biomarker for fetal vascular maturation ADC value consistent through gestational age therefore potential biomarker for postnatal renal function
T1 DIXON	Whole body Adpiose	Fat volume in the third trimester increases in fetuses of diabetic mothers, therefore potential biomarker for postnatal macrosomia

a murine model

In the lungs, T2* relaxation times and ADC values increase with gestational age, and the BOLD effect could be used as a marker for fetal oxygen uptake *in utero*. Perfusion fraction increases with gestational age and is reduced in the ipsilateral lung of fetuses with CDH. Together these could be used as a prenatal predictor of postnatal pulmonary hypoplasia.

In the liver, the T2* relaxation time is consistent throughout gestation and could be a marker for congenital haemochromatosis, informing immediate postnatal treatment. The T2* relaxation time reduces with hypoxia during Braxton-Hicks contractions, and the decreasing IVIM perfusion fraction could be used as a marker of maturation in conditions affecting the liver, such as CDH or giant omphalocoele.

In the kidney, the T2* relaxation time and ADC values remain consistent through gestational age, and the BOLD effect is observed in fetal renal ischaemia and maternal hypoxia. Therefore, together these are good targets for a prenatal imaging biomarkers of post-natal function

Finally, third trimester fat volume increases in fetuses of diabetic mothers and could act as a prenatal biomarker for macrosomia and its associated complications.

Although some of the biomarkers described can be used together with anatomical features in clinical practice, the majority are yet to be correlated with post-natal organ function in a quantifiable way.

A huge amount of work requiring international cooperation between specialist centres, utilising standardised sequence parameters across various vendors is needed to achieve representative baseline values, in healthy fetuses at multiple gestational ages.

Should it be achieved, the reliable prenatal prediction of postnatal outcome would prove indispensable for prenatal counselling, and with further development, functional MRI could replace more invasive prognostic interventions used in fetal maternal medicine.

## References

[1] Sørensen A, Peters D, Simonsen C, Pedersen M, Stausbøl-Grøn B, Christiansen OB, et al . Changes in human fetal oxygenation during maternal Hyperoxia as estimated by BOLD MRI. Prenat Diagn 2013; 33: 141–45. doi: 10.1002/pd.4025 23233459

[2] Hernando D, Levin YS, Sirlin CB, Reeder SB . Quantification of liver iron with MRI: state of the art and remaining challenges. J Magn Reson Imaging 2014; 40: 1003–21. doi: 10.1002/jmri.24584 24585403 PMC4308740

[3] Labranche R, Gilbert G, Cerny M, Vu KN, Soulières D, Olivié D, et al . Liver iron Quantification with MR imaging: A Primer for Radiologists. Radiographics 2018; 38: 392–412. doi: 10.1148/rg.2018170079 29528818

[4] Avena-Zampieri CL, Hutter J, Rutherford M, Milan A, Hall M, Egloff A, et al . Assessment of the fetal lungs in utero. Am J Obstet Gynecol MFM 2022; 4(): 100693. doi: 10.1016/j.ajogmf.2022.100693 35858660 PMC9811184

[5] Reeder SB, Pineda AR, Wen Z, Shimakawa A, Yu H, Brittain JH, et al . Iterative decomposition of water and fat with echo asymmetry and least-squares estimation (IDEAL): application with fast spin-echo imaging. Magn Reson Med 2005; 54: 636–44. doi: 10.1002/mrm.20624 16092103

[6] Yu H, Shimakawa A, McKenzie CA, Brodsky E, Brittain JH, Reeder SB . Multiecho water-fat separation and simultaneous R2* estimation with Multifrequency fat spectrum modeling. Magn Reson Med 2008; 60: 1122–34. doi: 10.1002/mrm.21737 18956464 PMC3070175

[7] Giza SA, Miller MR, Parthasarathy P, de Vrijer B, McKenzie CA . Comparison of modified two-point Dixon and chemical shift encoded MRI water-fat separation methods for fetal fat Quantification. J Magn Reson Imaging 2018; 48: 274–82. doi: 10.1002/jmri.25929 29319918

[8] Witzani L, Brugger PC, Hörmann M, Kasprian G, Csapone-Balassy C, Prayer D . Normal renal development investigated with fetal MRI. Eur J Radiol 2006; 57: 294–302. doi: 10.1016/j.ejrad.2005.11.027 16406436

[9] Le Bihan D, Breton E, Lallemand D, Grenier P, Cabanis E, Laval-Jeantet M . MR imaging of Intravoxel incoherent motions: application to diffusion and perfusion in neurologic disorders. Radiology 1986; 161: 401–7. doi: 10.1148/radiology.161.2.3763909 3763909

[10] Lemke A, Stieltjes B, Schad LR, Laun FB . Toward an optimal distribution of B values for Intravoxel incoherent motion imaging. Magn Reson Imaging 2011; 29: 766–76. doi: 10.1016/j.mri.2011.03.004 21549538

[11] Epstein SC, Bray TJP, Hall-Craggs MA, Zhang H . Task-driven assessment of experimental designs in diffusion MRI: A computational framework. PLoS One 2021; 16(): e0258442. doi: 10.1371/journal.pone.0258442 34624064 PMC8500429

[12] Vasylechko S, Malamateniou C, Nunes RG, Fox M, Allsop J, Rutherford M, et al . T2* Relaxometry of fetal brain at 1.5 Tesla using a motion tolerant method. Magn Reson Med 2015; 73: 1795–1802. doi: 10.1002/mrm.25299 25046845

[13] Goitein O, Eshet Y, Hoffmann C, Raviv-Zilka L, Salem Y, Hamdan A, et al . Fetal liver T2* values: defining a standardized scale. J Magn Reson Imaging 2013; 38: 1342–45. doi: 10.1002/jmri.24132 23576455

[14] Sethi S, Giza SA, Goldberg E, Empey MET, Ribaupierre S, Eastabrook GDM, et al . Quantification of 1.5 T T1 and T2* relaxation times of fetal tissues in uncomplicated pregnancies. J Magn Reson Imaging 2021; 113–21. doi: 10.1002/jmri.27547 33586269

[15] Strobel KM, Kafali SG, Shih S-F, Artura AM, Masamed R, Elashoff D, et al . Pregnancies complicated by gestational diabetes and fetal growth restriction: an analysis of maternal and fetal body composition using magnetic resonance imaging. J Perinatol 2023; 43: 44–51. doi: 10.1038/s41372-022-01549-5 36319757 PMC9840659

[16] Jakab A, Tuura R, Kottke R, Kellenberger CJ, Scheer I . Intra-Voxel incoherent motion MRI of the living human Foetus: technique and test-retest Repeatability. Eur Radiol Exp 2017; 1: 26. doi: 10.1186/s41747-017-0031-4 29708192 PMC5909359

[17] Uus AU, Egloff Collado A, Roberts TA, Hajnal JV, Rutherford MA, Deprez M . Retrospective motion correction in Foetal MRI for clinical applications: existing methods, applications and integration into clinical practice. Br J Radiol 2022: 20220071. doi: 10.1259/bjr.20220071 35834425 PMC7614695

[18] Cannie M, Jani J, De Keyzer F, Roebben I, Dymarkowski S, Deprest J . Diffusion-weighted MRI in lungs of normal fetuses and those with congenital diaphragmatic hernia. Ultrasound Obstet Gynecol 2009; 34: 678–86. doi: 10.1002/uog.7326 19866446

[19] Dütemeyer V, Cordier A-G, Cannie MM, Bevilacqua E, Huynh V, Houfflin-Debarge V, et al . Prenatal prediction of postnatal survival in fetuses with congenital diaphragmatic hernia using MRI: lung volume measurement, signal intensity ratio, and effect of experience. J Matern Fetal Neonatal Med 2022; 35: 1036–44. doi: 10.1080/14767058.2020.1740982 32212880

[20] Terui K, Omoto A, Osada H, Hishiki T, Saito T, Sato Y, et al . Prediction of postnatal outcomes in congenital diaphragmatic hernia using MRI signal intensity of the fetal lung. J Perinatol 2011; 31: 269–73. doi: 10.1038/jp.2010.119 21052047

[21] Yamoto M, Iwazaki T, Takeuchi K, Sano K, Fukumoto K, Takahashi T, et al . The fetal lung-to-liver signal intensity ratio on magnetic resonance imaging as a Predictor of outcomes from isolated congenital diaphragmatic hernia. Pediatr Surg Int 2018; 34: 161–68. doi: 10.1007/s00383-017-4184-2 29018962

[22] Khen-Dunlop N, Chalouhi G, Lecler A, Bouchouicha A, Millischer A-E, Tavitian B, et al . Assessment of BOLD response in the fetal lung. Eur Radiol 2021; 31: 3090–97. doi: 10.1007/s00330-020-07272-z 33123792

[23] Avena-ZampieriCL, HutterJ, DeprezM, PayetteK, HallM, UusA, et al . Assessment of normal pulmonary development using functional magnetic resonance imaging techniques. Am J Obstet Gynecol MFM 2023; 5: 100935. doi: 10.1016/j.ajogmf.2023.100935 36933803

[24] Balassy C, Kasprian G, Brugger PC, Csapo B, Weber M, Hörmann M, et al . Diffusion-weighted MR imaging of the normal fetal lung. Eur Radiol 2008; 18: 700–706. doi: 10.1007/s00330-007-0784-x 17924118

[25] Manganaro L, Perrone A, Sassi S, Fierro F, Savelli S, Di Maurizio M, et al . Diffusion-weighted MR imaging and apparent diffusion coefficient of the normal fetal lung: preliminary experience. Prenat Diagn 2008; 28: 745–48. doi: 10.1002/pd.2041 18567059

[26] Afacan O, Gholipour A, Mulkern RV, Barnewolt CE, Estroff JA, Connolly SA, et al . Fetal lung apparent diffusion coefficient measurement using diffusion-weighted MRI at 3 Tesla: correlation with gestational age. J Magn Reson Imaging 2016; 44: 1650–55. doi: 10.1002/jmri.25294 27159847 PMC5436687

[27] Moradi B, Ghorbani Z, Shirazi M, Gity M, Kazemi MA, Sharifian H, et al . Comparison of fetal lung maturation in fetuses with Intrauterine growth restriction with control group, using lung volume, lung/liver and lung/muscle signal intensity and apparent diffusion coefficient ratios on different magnetic resonance imaging sequences. J Matern Fetal Neonatal Med 2022; 35: 8936–44. doi: 10.1080/14767058.2021.2008349 34847801

[28] Prayer F, Watzenböck ML, Heidinger BH, Rainer J, Schmidbauer V, Prosch H, et al . Fetal MRI Radiomics: non-invasive and reproducible Quantification of human lung maturity. Eur Radiol 2023; 33: 4205–13. doi: 10.1007/s00330-022-09367-1 36604329 PMC10182107

[29] Ronkainen E, Dunder T, Kaukola T, Marttila R, Hallman M . Intrauterine growth restriction predicts lower lung function at school age in children born very Preterm. Arch Dis Child Fetal Neonatal Ed 2016; 101: F412–7. doi: 10.1136/archdischild-2015-308922 26802110

[30] Greenough A, Yuksel B, Cheeseman P . Effect of in utero growth retardation on lung function at follow-up of prematurely born infants. Eur Respir J 2004; 24: 731–33. doi: 10.1183/09031936.04.00060304 15516664

[31] Jakab A, Tuura RL, Kottke R, Ochsenbein-Kölble N, Natalucci G, Nguyen TD, et al . Microvascular perfusion of the Placenta, developing fetal liver, and lungs assessed with Intravoxel incoherent motion imaging. J Magn Reson Imaging 2018; 48: 214–25. doi: 10.1002/jmri.25933 29281153

[32] Ercolani G, Capuani S, Antonelli A, Camilli A, Ciulla S, Petrillo R, et al . Intravoxel incoherent motion (IVIM) MRI of fetal lung and kidney: can the perfusion fraction be a marker of normal pulmonary and renal maturation? Eur J Radiol 2021; 139: 109726. doi: 10.1016/j.ejrad.2021.109726 33895624

[33] Wynn R, Bhat R, Monagle P . Pediatric Hematology. In: Normal hematopoiesis and the physiology of blood. Pediatric hematology: A practical guide. Cambridge: Cam- bridge University Press; 16 February 2017., pp. 1–6. doi: 10.1017/CBO9781139942430

[34] Mikkola HKA, Orkin SH . The journey of developing hematopoietic stem cells. Development 2006; 133: 3733–44. doi: 10.1242/dev.02568 16968814

[35] Muckenthaler MU, Rivella S, Hentze MW, Galy B . A red carpet for iron metabolism. Cell 2017; 168: 344–61. doi: 10.1016/j.cell.2016.12.034 28129536 PMC5706455

[36] Barzin M, Kowsarian M, Akhlaghpoor S, Jalalian R, Taremi M . Correlation of cardiac MRI T2* with echocardiography in Thalassemia major. Eur Rev Med Pharmacol Sci 2012; 16: 254–60. 22428478

[37] Abaci Turk E, Stout JN, Feldman HA, Gagoski B, Zhou C, Tamen R, et al . Change in T2* measurements of Placenta and fetal organs during Braxton Hicks contractions. Placenta 2022; 128: 69–71. doi: 10.1016/j.placenta.2022.08.011 36087451 PMC9674925

[38] Yushkevich PA, Piven J, Hazlett HC, Smith RG, Ho S, Gee JC, et al . User-guided 3D active contour Segmentation of anatomical structures: significantly improved efficiency and reliability. Neuroimage 2006; 31: 1116–28. doi: 10.1016/j.neuroimage.2006.01.015 16545965

[39] Hansen DN, Sinding M, Petersen A, Christiansen OB, Uldbjerg N, Peters DA, et al . T2*-Weighted Placental magnetic resonance imaging: a biomarker of Placental dysfunction in small-for-gestational-age pregnancies. Am J Obstet Gynecol MFM 2022; 4: 100578. doi: 10.1016/j.ajogmf.2022.100578 35114424

[40] Morris DM, Ross JA, McVicar A, Semple SIK, Haggarty P, Gilbert FJ, et al . Changes in Foetal liver T2* measurements by MRI in response to maternal oxygen breathing: application to diagnosing Foetal growth restriction. Physiol Meas 2010; 31: 1137–46. doi: 10.1088/0967-3334/31/9/005 20651423

[41] Chalouhi GE, Millischer A-É, Mahallati H, Siauve N, Melbourne A, Grevent D, et al . The use of fetal MRI for renal and Urogenital tract anomalies. Prenat Diagn 2020; 40: 100–109. doi: 10.1002/pd.5610 31736096

[42] Manganaro L, Francioso A, Savelli S, Tomei A, Fierro F, Di Maurizio M, et al . Fetal MRI with diffusion-weighted imaging (DWI) and apparent diffusion coefficient (ADC) assessment in the evaluation of renal development: preliminary experience in normal kidneys. Radiol Med 2009; 114: 403–13. doi: 10.1007/s11547-009-0382-x 19381763

[43] Savelli S, Di Maurizio M, Perrone A, Tesei J, Francioso A, Angeletti M, et al . MRI with diffusion-weighted imaging (DWI) and apparent diffusion coefficient (ADC) assessment in the evaluation of normal and abnormal fetal kidneys: preliminary experience. Prenat Diagn 2007; 27: 1104–11. doi: 10.1002/pd.1839 17849498

[44] Chaumoitre K, Colavolpe N, Shojai R, Sarran A, D’ Ercole C, Panuel M . Diffusion-weighted magnetic resonance imaging with apparent diffusion coefficient (ADC) determination in normal and pathological fetal kidneys. Ultrasound Obstet Gynecol 2007; 29: 22–31. doi: 10.1002/uog.3892 17167818

[45] Faure A, Panait N, Panuel M, Alessandrini P, D’Ercole C, Chaumoitre K, et al . Predicting postnatal renal function of prenatally detected posterior Urethral valves using fetal diffusion-weighted magnetic resonance imaging with apparent diffusion coefficient determination. Prenat Diagn 2017; 37: 666–72. doi: 10.1002/pd.5063 28453880

[46] Giza SA, Olmstead C, McCooeye DA, Miller MR, Penava DA, Eastabrook GD, et al . Measuring fetal Adipose tissue using 3D water-fat magnetic resonance imaging: a feasibility study. J Matern Fetal Neonatal Med 2020; 33: 831–37. doi: 10.1080/14767058.2018.1506438 30189758

[47] Anblagan D, Deshpande R, Jones NW, Costigan C, Bugg G, Raine-Fenning N, et al . Measurement of fetal fat in utero in normal and diabetic pregnancies using magnetic resonance imaging. Ultrasound Obstet Gynecol 2013; 42: 335–40. doi: 10.1002/uog.12382 23288811

[48] Berger-Kulemann V, Brugger PC, Reisegger M, Klein K, Hachemian N, Koelblinger C, et al . Quantification of the subcutaneous fat layer with MRI in fetuses of healthy mothers with no underlying metabolic disease vs. fetuses of diabetic and obese mothers. J Perinat Med 2011; 40: 179–84. doi: 10.1515/JPM.2011.122 22117112

[49] Friesen-Waldner LJ, Sinclair KJ, Wade TP, Michael B, Chen AP, de Vrijer B, et al . Hyperpolarized [1-(13) C]Pyruvate MRI for noninvasive examination of Placental metabolism and nutrient transport: A feasibility study in pregnant Guinea pigs. J Magn Reson Imaging 2016; 43: 750–55. doi: 10.1002/jmri.25009 26227963

[50] Nelson TR, Gillies RJ, Powell DA, Schrader MC, Manchester DK, Pretorius DH . High resolution proton NMR spectroscopy of human Amniotic fluid. Prenat Diagn 1987; 7: 363–72. doi: 10.1002/pd.1970070511 3615362

[51] Groenen PMW, Engelke UF, Wevers RA, Hendriks JCM, Eskes TKAB, Merkus HMWM, et al . High-resolution 1H NMR spectroscopy of Amniotic fluids from Spina Bifida fetuses and controls. Eur J Obstet Gynecol Reprod Biol 2004; 112: 16–23. doi: 10.1016/s0301-2115(03)00279-3 14687733

[52] Amorini AM, Giorlandino C, Longo S, D’Urso S, Mesoraca A, Santoro ML, et al . Metabolic profile of Amniotic fluid as a biochemical tool to screen for inborn errors of metabolism and fetal anomalies. Mol Cell Biochem 2012; 359: 205–16. doi: 10.1007/s11010-011-1015-y 21837404

[53] Naila D, Sadanand S, Sussman D . Artificial Amniotic fluid for nuclear magnetic resonance spectroscopy studies. Anal Sci Adv 2022; 3: 174–87. doi: 10.1002/ansa.202100055 PMC10989604 38716122

[54] Prayer D, Malinger G, De Catte L, De Keersmaecker B, Gonçalves LF, Kasprian G, et al . ISUOG practice guidelines (updated): performance of fetal magnetic resonance imaging. Ultrasound Obstet Gynecol 2023; 61: 278–87. doi: 10.1002/uog.26129 36722431 PMC10107509

